# Comparison of glucose concentrations in simultaneously collected plasma and serum samples from outpatients in a routine laboratory setting

**DOI:** 10.1371/journal.pone.0344562

**Published:** 2026-03-10

**Authors:** Robert Müller, Diethard Müller, Dietmar Plonné

**Affiliations:** 1 MVZ Labor Ravensburg SE and Co., eGbR, Limbach Gruppe, Ravensburg, Germany; 2 Private University of the Principality of Liechtenstein (UFL), Triesen, Liechtenstein; University of Toronto Department of Laboratory Medicine and Pathobiology, CANADA

## Abstract

**Objectives:**

Although international and national German guidelines recommend measuring glucose in venous plasma, serum remains the most commonly used sample type for glucose measurement in routine outpatient settings in Germany. The aim of the study was to compare glucose concentrations in simultaneously collected serum and plasma samples from outpatients under routine conditions, and to assess whether any observed differences could lead to divergent diagnostic outcomes for diabetes mellitus.

**Methods:**

Between 03/01/2022 and 20/12/2024, a total of 27,864 simultaneously collected sodium fluoride plasma (NaF plasma) and serum sample pairs were submitted by 200 different medical practices to MVZ Labor Ravensburg for glucose measurement. Serum samples, all containing separator gel, were centrifuged at the medical practice after complete coagulation. In contrast, NaF plasma samples were transported uncentrifuged. Upon arrival at the laboratory, all samples were centrifuged immediately and analyzed within two hours. The median of the differences between serum and plasma glucose was calculated for each quintile of the mean glucose concentration. A contingency table was used to analyze the classification outcomes based on plasma and serum glucose concentrations.

**Results:**

In the concentration range above 100 mg/dL, the difference between serum and plasma glucose levels was minimal (4th quintile: 0.54 mg/dL; 5th quintile: 1.13 mg/dL). Below 100 mg/dL, the difference increased linearly with decreasing glucose concentration (3rd quintile: −1.69 mg/dL; 2nd quintile: −5.20 mg/dL; 1st quintile: −11.05 mg/dL). Using 126 mg/dL as the glucose cutoff, positive and negative diabetes classifications from NaF plasma and serum glucose agreed in 97.8% of cases. Overall, only 0.3% more cases of diabetes mellitus were classified based on NaF plasma glucose compared to serum glucose.

**Conclusions:**

In real-world outpatient settings, glucose measurement in on-site centrifuged serum with separator gel does not appear to present a clinically relevant disadvantage for diabetes mellitus screening compared to on-site uncentrifuged NaF plasma. However, neither serum nor non-acidified NaF plasma ensures complete inhibition of in vitro glycolysis and must therefore be regarded as suboptimal for definitive diagnostic purposes.

## Introduction

Glucose is one of the most commonly requested analytes in clinical chemistry laboratories. Accurate and reliable measurement of glucose concentration is essential for the diagnosis and monitoring of diabetes mellitus, gestational diabetes, impaired glucose tolerance, and impaired fasting glucose [1]. The most significant preanalytical error in glucose analysis is the *in vitro* glycolytic degradation of glucose by blood cells, particularly erythrocytes. This can lead to falsely low glucose values and, consequently, to the underdiagnosis of a diabetic metabolic state. The rate of glycolysis is influenced by several factors, including how quickly plasma is separated from blood cells, ambient temperature, white blood cell count, and glucose concentration [2–4]. Several strategies have been proposed to overcome this critical preanalytical problem. Glycolysis can be inhibited by different additives, such as sodium fluoride, typically used in combination with the anticoagulants EDTA (NaF/EDTA) or heparin (NaF/heparin), or more effectively by acidifying the blood with a citrate buffer together with sodium fluoride and EDTA (citrate/fluoride/EDTA). Because the inhibition of the late glycolytic enzyme enolase by sodium fluoride (NaF) is insufficient to prevent glycolysis in the first few hours after sample collection [5], the recommended additive is citrate/fluoride/EDTA. This combination not only inhibits enolase but also immediately blocks the early glycolytic enzyme hexokinase, thereby effectively preventing glycolysis [2,6,7]. In the absence of glycolysis-inhibiting additives, glucose degradation can be minimized by cooling the samples and performing rapid centrifugation to remove the cells within 15–30 minutes [8,9]. Although clinical guidelines do not recommend using serum or non-acidified NaF-containing plasma for diagnosing diabetes mellitus [2,10], these sample types remain the most commonly used for diabetes screening in the outpatient setting in Germany. This is primarily due to cost and practicality, as NaF/EDTA or NaF/heparin tubes are still less expensive than the recommended citrate/NaF/EDTA tubes. An additional advantage of using serum is that, apart from glucose, numerous other laboratory parameters can be measured from a single blood sample, saving time and costs while also reducing the burden on the patient. Regarding the discrepancies in glucose levels between plasma and serum, some studies have reported that plasma glucose is higher than serum glucose, while others have found no significant difference [11]. However, most studies comparing glucose levels in plasma and serum samples have been conducted under strictly controlled laboratory conditions that do not reflect the real-world settings of routine medical practice and clinical laboratories.

Therefore, the aim of the present study was to investigate the extent to which glucose concentrations differ between plasma and serum samples collected simultaneously from outpatients under real-world conditions, and to assess whether any such differences could influence the diagnosis of diabetes mellitus. This study was not designed to investigate the impact of pre-analytical factors on differences between plasma and serum glucose concentrations under controlled laboratory conditions. Accordingly, the present study does not challenge current guideline recommendations but rather evaluates the clinical impact of routinely used sample types under real-world outpatient conditions.

## Materials and methods

### Ethics statement

The study protocol was reviewed by the Ethics Committee of the State Medical Association of Baden-Württemberg (Germany) and approved without objections (reference F-2024-006). The study used anonymized routine laboratory data only and was conducted in accordance with the principles of the Declaration of Helsinki. Informed consent was waived due to the exclusive use of anonymized data.

### Collection of plasma and serum samples

Between 03/01/2022 and 20/12/2024, a total of 27,864 simultaneously collected venous plasma and serum samples from 12,869 men and 14,995 women were submitted to MVZ Labor Ravensburg for glucose measurement. The samples were obtained from 200 medical practices specializing in the following areas: general medicine, internal medicine, orthopedics, occupational medicine, cardiology, neurology, gynecology and obstetrics, psychiatry. All data were anonymized prior to analysis, ensuring that no identification of individual patients or referring physicians was possible. Based on the approved ethics application, the data were accessed for analysis starting on 26/02/2024.

Blood samples were typically collected in the morning. However, the exact time of collection was not documented. No information was available regarding the clinical diagnosis, indication for testing, or the patient’s fasting status. After blood collection and complete coagulation, serum tubes containing separating gel were centrifuged (Hettich, Eba 200; 3,000 × g for 10 minutes) on-site in the medical practice to separate the serum from the clot and blood cells. Although practice staff were trained to perform centrifugation within 30–60 minutes after blood collection, the exact time interval between collection and centrifugation was not documented. In contrast to the serum samples, the NaF plasma samples were not centrifuged on-site.

No information was available regarding the storage conditions of the samples at the medical practice prior to collection by the laboratory courier service. Transport of the samples from the medical practice to the laboratory was performed by accredited courier services under controlled temperature conditions (8–25 °C) and typically took 2–6 hours. Upon arrival at the laboratory, both plasma and serum samples were centrifuged at 3,000 × g for 10 minutes at 20 °C (Hettich, Rotixa 500 RS, Tuttlingen, Germany), and glucose concentrations were subsequently measured. The maximum time interval between serum and the corresponding plasma measurements was three hours, with a median interval of 0.33 hours.

### Inclusion criteria

For the statistical analysis, 27,328 plasma/serum sample pairs from 12,603 men and 14,725 women were included. This represented 98.1% of all incoming sample pairs that met the following inclusion criteria: age between 18 and 95 years, glucose concentrations between 40 and 300 mg/dL, and a maximum deviation between plasma and serum glucose values of ±50 mg/dL. Extreme values outside the predefined inclusion criteria, accounting for 0.7% of all observations, were excluded to allow for an informative graphical representation of the data on a linear scale. This exclusion did not affect the medians of plasma and serum glucose concentrations or the median difference between plasma and serum glucose.

### Plasma and serum tubes

The following tube types were used for the determination of glucose in serum:

- Sarstedt S-Monovette® Serum Gel CAT, 7.5 mL, cap brown, (L x Ø): 92 x 15 mm, with plastic label, order number: 01.1602 (Sarstedt, Nümbrecht, Germany)
- BD Vacutainer™ SST™ II Advance Tubes, Brand: BD 367953 (Becton Dickinson, Heidelberg, Germany)

The following tube types were used for the determination of glucose in plasma:

- Sarstedt S-Monovette® Fluoride/EDTA FE, 2.7 mL, cap yellow, (L x Ø): 75 x 13 mm, with plastic label, order number: 04.1918 (Sarstedt, Nümbrecht, Germany)
- BD® Vacutainer® FH 20 mg 143 IU Blood Collection Tube, 5.0 mL, Brand: BD 367764 (Becton Dickinson, Heidelberg, Germany)

The listed tube types were used in approximately equal proportions. NaF plasma refers to plasma samples collected in sodium fluoride–containing tubes, using either EDTA or heparin as the anticoagulant.

### Measurement of glucose

Glucose measurement was performed on the ADVIA 2400 analyzer (Siemens, Eschborn, Germany) using the glucose hexokinase method (reagent name: ADVIA® Chemistry Glucose Hexokinase_3 Concentrated [GLUH_c]). Glucose measurement was performed on a randomly selected analyzer from a total of six available analyzers, with the samples of each pair analyzed separately.

### Statistical analysis and data visualization

Statistical analyses and graphical visualizations were performed using R statistical software (version 4.5.0; R Core Team, 2024) and RStudio (2025.05.0 Build 496). Figures were generated using the ggplot2 package, with additional analyses supported by the ggstatsplot [12], DescTools [13], and epiR [14] packages. Distributions of plasma and serum glucose concentrations were examined using nonparametric kernel density estimation. Agreement between paired plasma and serum glucose measurements was explored using scatter plots and difference plots. A locally weighted smoothing curve (LOESS regression) was applied to the difference plot to explore potential concentration-dependent bias. Differences between sample types were additionally examined across quintiles of mean glucose concentration and stratified by calendar quarter (Q1–Q4) to assess potential seasonal effects related to colder and warmer periods. Diagnostic accuracy for the binary classification of diabetes mellitus at a glucose cutoff of 126 mg/dL was assessed by estimating sensitivity and specificity with exact 95% confidence intervals, and agreement between plasma- and serum-based classifications was evaluated using Cohen’s kappa statistic. A statistically significant difference was considered present if the 95% confidence intervals of two values did not overlap. The 95% confidence intervals are reported in square brackets.

## Results

The mean age was 58.2 ± 15.9 years (mean ± SD) for males and 58.9 ± 16.4 years for females.

**Fig 1:**
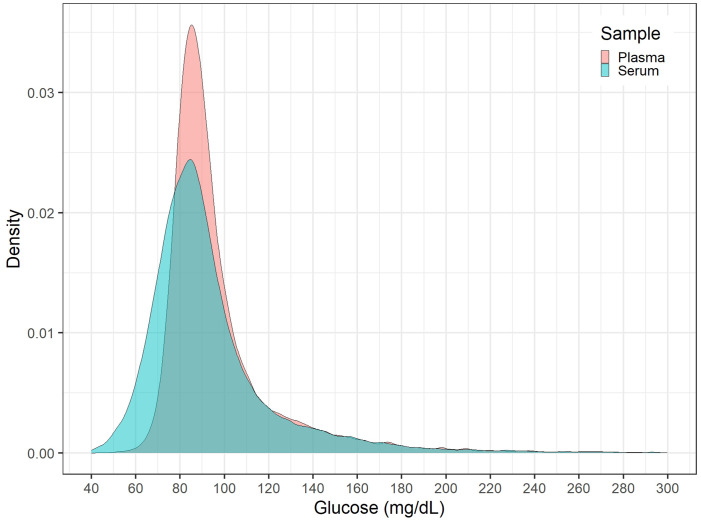
Density plot showing the distribution of glucose concentrations in 27,328 paired plasma and serum samples.

Plasma and serum glucose concentrations exhibited a right-skewed distribution, with the plasma and serum curves nearly overlapping in the upper range above 115 mg/dL (Fig 1).

Conversely, a leftward shift of the serum curve relative to the plasma curve is evident at lower glucose concentrations, indicating a higher frequency of low glucose values in serum compared to plasma samples. Due to the skewed distribution of glucose values, the arithmetic mean was higher than the corresponding median by 8.2 mg/dL in plasma and by 6.7 mg/dL in serum (see row M + F in Table 1). The median glucose concentration was significantly higher in males than in females by 4.0 mg/dL in plasma and 3.2 mg/dL in serum (Table 1).

**Table 1:** Statistical measures of glucose concentrations (mg/dL) in paired plasma and serum samples.

Sex	n	Sample	Median	95% CI	Minimum	Maximum	Mean	95% CI
M + F	27,328	Plasma	89.6	89.4–89.8	42.4	300.0	97.8	97.4–98.1
		Serum	86.7	86.4–86.9	40.0	289.9	93.4	93.1–93.8
M	12,603	Plasma	91.9	91.6–92.2	45.6	299.9	101.1	100.5–101.6
		Serum	88.5	88.1–88.9	40.0	298.9	96.1	95.5–96.7
F	14,725	Plasma	87.9	87.6–88.1	42.4	300.0	94.9	94.5–95.3
		Serum	85.3	85.0–85.6	40.2	298.1	91.2	90.7–91.6

M, Male; F, Female; n, number of observations; CI, confidence interval.

The median plasma glucose concentration was 3.4 mg/dL higher than the median serum glucose in men and 2.6 mg/dL higher in women, resulting in a sex-related difference in the plasma–serum gap of 0.8 mg/dL. Given the small difference, data from both sexes were combined for further comparisons of plasma and serum glucose in paired samples.

**Fig 2:**
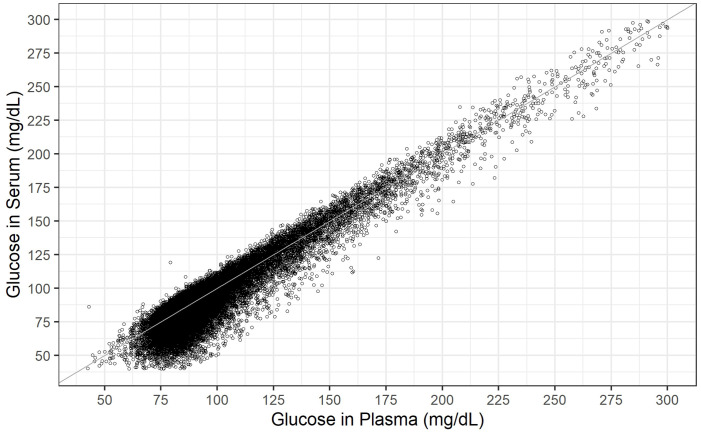
Scatter plot showing glucose concentrations in simultaneously collected plasma and serum samples (n = 27,328). The gray line represents the line of identity (y = **x)**. Pearson correlation coefficient = 0.945 [95% CI: 0.943 to 0.946]; Lin’s concordance correlation coefficient = 0.931 [95% CI: 0.929 to 0.932].

As shown in the scatter plot (Fig 2), there was a strong positive correlation between plasma and serum glucose concentrations.

**Fig 3:**
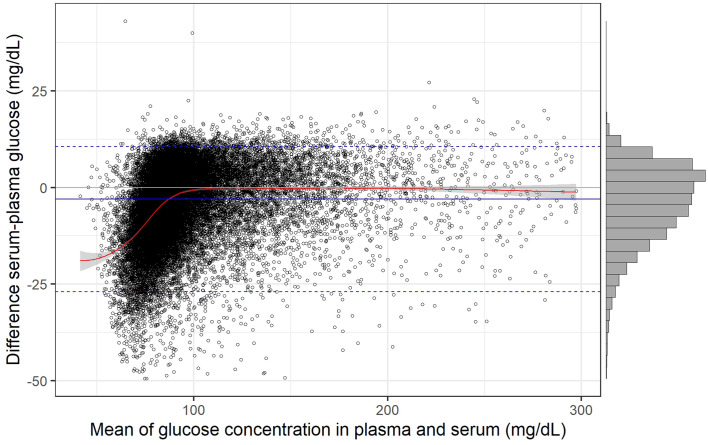
Difference plot with histogram showing the absolute differences between serum glucose and plasma glucose concentrations plotted against their mean. The median difference is indicated by the solid blue line, and the 95% limits of agreement are shown as dotted blue lines. The red solid line represents the LOESS regression line, with its 95% confidence band shown in gray. Median difference = −3.05 [95% CI: −3.23 to −2.89]; 95% lower limit of agreement = −26.98 [95% CI: −27.40 to −26.62]; 95% upper limit of agreement = 10.54 [95% CI: 10.38 to 10.71].

The scatter plot points were nearly symmetrically distributed around the identity line at higher glucose concentrations (>125 mg/dL). However, in the lower concentration range, the distribution was asymmetric, with serum values more frequently falling below plasma values. This slight systematic bias was reflected in a lower Lin’s concordance correlation coefficient [15] of 0.931 (95% CI: 0.929–0.932) relative to Pearson’s correlation coefficient of 0.945 (95% CI: 0.943–0.946), indicating moderate agreement (0.90–0.95) between the two measurements according to McBride [16]. The asymmetry in the deviation from the identity line is more clearly illustrated in the difference plot (Fig 3), where the absolute difference between serum and plasma glucose concentrations is plotted against the mean of the two measurements. The histogram in Fig 3 demonstrated a skewed distribution of the differences, making the median more appropriate than the arithmetic mean for statistical analysis. Across the entire concentration range, the median difference between serum and plasma glucose concentrations (serum minus plasma) was −3.1 mg/dL [95% CI: −3.2 to −2.9]. The 95% range of differences extended from −27.0 mg/dL [95% CI: −27.4 to −26.6] to 10.5 mg/dL [95% CI: 10.4 to 10.7]. In the lower concentration range (approximately <100 mg/dL), the locally estimated scatterplot smoothing (LOESS) regression curve showed an almost linear relationship between the serum–plasma glucose difference and the mean glucose concentration, with the difference increasing as the mean concentration decreased. Above 100 mg/dL, however, the LOESS regression curve was nearly identical to the zero line, indicating no substantial difference between serum and plasma glucose concentrations within this range.

**Fig 4:**
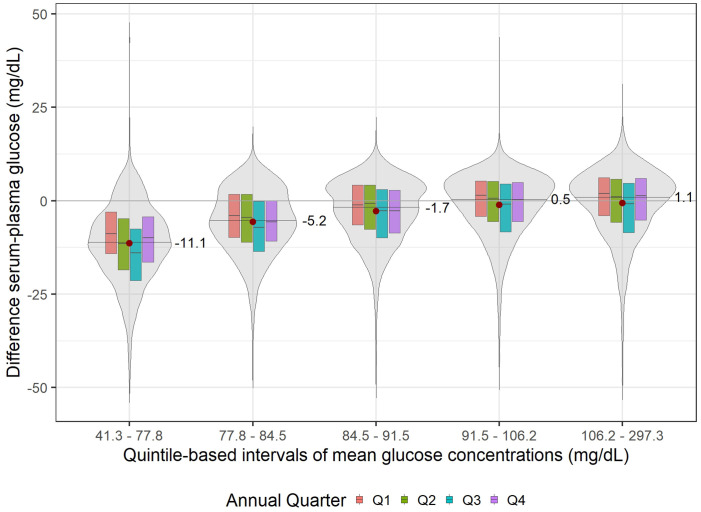
Violin plot of serum–plasma differences in glucose concentration (y-axis) across quintiles of mean glucose concentration (x-axis). The horizontal black line within each violin represents the median difference, with corresponding numerical values. The red dot denotes the arithmetic mean. Box plots embedded within the violins illustrate the distribution of differences by annual quarter (Q1, Q2, Q3, Q4) within each quintile.

For a quantitative analysis of the differences between plasma and serum glucose at varying glucose concentrations, the mean glucose values were grouped into quintiles, and the median and mean differences were calculated (Fig 4).

The largest median negative difference between serum and plasma glucose was −11.05 mg/dL [95% CI: −11.3 to −10.7] in the first quintile (41.3–77.8 mg/dL, n = 5,463), followed by −5.2 mg/dL [95% CI: −5.5 to −4.9] in the second quintile (77.8–84.5 mg/dL, n = 5,468) and −1.7 mg/dL [95% CI: −2.0 to −1.4] in the third quintile (84.5–91.5 mg/dL, n = 5,465). Therefore, at mean glucose concentrations below 100 mg/dL, the serum–plasma glucose difference increased by approximately 3.2 mg/dL for every 10 mg/dL decrease in the mean glucose concentration. In the fourth quintile (91.5–106.2 mg/dL, n = 5,466) and the fifth quintile (106.2–297.3 mg/dL, n = 5,466), the median differences were the smallest, measuring 0.5 mg/dL [95% CI: 0.3 to 0.8] and 1.1 mg/dL [95% CI: 0.9 to 1.4], respectively. As shown by the box plots for each annual quarter within the violin plots, the serum–plasma glucose difference varied seasonally, with the largest difference observed in the summer quarter (Q3) and smaller differences during the colder seasons (Q1, Q2, and Q4). The largest serum–plasma glucose differences were observed between Q1 and Q3 across all quintiles, with values of −5.1 mg/dL, −3.1 mg/dL, −1.6 mg/dL, −2.4 mg/dL, and −2.8 mg/dL for quintiles 1, 2, 3, 4, and 5, respectively (S1 Table).

The diagnostic cutoff for diabetes mellitus is a fasting glucose level of ≥126 mg/dL [2]. To determine whether differences in glucose concentrations between serum and plasma would lead to different diagnostic outcomes, glucose values <126 mg/dL were classified as negative (neg), and values ≥126 mg/dL as positive (pos) for diabetes mellitus, separately for serum and plasma. The results are presented in the contingency table (Table 2).

**Table 2:** Contingency table showing the number and percentage of samples classified with a hypothetical diagnosis of diabetes mellitus based on glucose concentrations measured in plasma and serum. Glucose concentrations <126 mg/dL were classified as negative (neg) and concentrations ≥126 mg/dL as positive (pos).

Plasma	Serum		
	Neg	Pos	Total
**Neg**	**24,077** (88.1%)	**260** (1.0%)	24,337 (89.1%)
**Pos**	**352** (1.3%)	**2,639** (9.7%)	2,991 (10.9%)
**Total**	24,429 (89.4%)	2,899 (10.6%)	27,328 (100%)

Point estimates with 95% CI
  Cohen‘s kappa                         0.884 [95% CI: 0.874 to 0.893]
  Correctly classified proportion                   97.8% [95% CI: 97.4 to 98.1]
  Agreement of positive results                   98.9% [95% CI: 98.8 to 99.1]
  Agreement of negative results                   88.2% [95% CI: 87.0 to 89.3]
  Plasma positive percent proportion for total serum negative results   1.4% [95% CI: 1.3 to 1.6]
  Serum positive percent proportion for total plasma negative results   1.1% [95% CI: 0.9 to 1.2]
  Proportion difference of positive results               0.3% [95% CI: 0.2 to 0.5]

The calculated Cohen’s kappa was 0.884 [95% CI: 0.874 to 0.893], which can be interpreted as almost perfect agreement [17]. Overall, 97.8% [95% CI: 97.4 to 98.1] of the classifications based on plasma and serum glucose values were consistent. The agreement of serum with plasma was 98.9% [95% CI: 98.8 to 99.1] for the positive classification and 88.2% [95% CI: 87.0 to 89.3] for the negative classification. A total of 1.1% [95% CI: 0.9 to 1.2] of negative plasma results were positive in serum, and 1.4% [95% CI: 1.3 to 1.6] of negative serum results were positive in plasma. This indicates that 0.3% more cases of diabetes mellitus [95% CI: 0.2 to 0.5] would have been diagnosed based on plasma rather than serum values.

## Discussion

Under real-world conditions, we observed lower glucose concentrations in serum than in plasma, predominantly in the lower concentration range (<100 mg/dL). In the range above 100 mg/dL, differences between serum and plasma glucose concentrations were statistically significant but of minor clinical relevance. Based on the diagnostic cutoff value of 126 mg/dL for diabetes mellitus screening, glucose measurement in serum would detect 3 fewer pathological cases per 1,000 measurements than measurement in plasma. Given that a single laboratory value is not sufficient for the diagnosis of diabetes mellitus [2], this difference appears to be negligible. According to national guidelines for type 2 diabetes, the diagnosis of diabetes mellitus is confirmed if two laboratory results – such as fasting plasma glucose and HbA1c, fasting plasma glucose and occasional plasma glucose, two fasting plasma glucose measurements, or HbA1c and occasional plasma glucose – are within the pathological range [2]. If the results are inconsistent, a third laboratory test should be performed, and if necessary, an oral glucose tolerance test (oGTT) should be conducted. The guidelines recommend the use of venous peripheral plasma rather than serum for measuring glucose for diagnosing diabetes mellitus. Plasma is the preferred specimen because *in vitro* glycolysis as the most significant pre-analytical source of error can be effectively minimized by rapid centrifugation of the blood sample immediately after blood collection and/or by the addition of glycolysis inhibitors. In uncentrifuged specimens, the decrease in glucose concentration during the first two hours after blood collection is nearly identical in serum and non-acidified NaF plasma, amounting to approximately 4–5% per hour at room temperature [18]. Thereafter, serum glucose concentration continues to decline, while glucose levels in NaF plasma remain stable. An immediate and complete stop of *in vitro* glycolysis can be achieved by adding citrated NaF/EDTA, eliminating the need for centrifugation [19–22]. This is because in citrated NaF/EDTA plasma, in addition to the inhibition of the late glycolytic enzyme enolase, the initial enzyme of glycolysis, hexokinase, is also inhibited, thereby preventing glycolysis at a much earlier stage [23]. However, unlike conventional NaF tubes, citrated NaF/EDTA tubes are not yet widely used for glucose measurement in the outpatient setting in Germany. In our comparative study, only NaF tubes without the addition of citrate were used. Although all practices that submitted blood samples are equipped with centrifuges, the NaF plasma was not centrifuged on-site due to practical considerations. NaF tubes do not contain a separation gel. Therefore, reliable separation of blood cells from plasma cannot be guaranteed, particularly during transport to the laboratory. Alternatively, pipetting plasma into a new tube after centrifugation is not practical within the typical workflow of a medical practice. In contrast to the NaF tubes, the serum tubes used contain a separation gel that ensures reliable separation of blood cells from serum, even during transport. Therefore, the serum tubes were centrifuged on-site after the samples had fully coagulated. Although the practice staff were trained to centrifuge serum samples between 20 and 60 minutes after venipuncture, the actual time elapsed between sampling and centrifugation was not recorded. Because centrifugation of the NaF tubes did not occur in the physician’s practice but rather in the laboratory, with a delay of several hours, the glucose concentration could have decreased by up to 10 mg/dL due to the delayed inhibition of glycolysis by NaF [2]. Furthermore, inadequate mixing of the blood immediately after sampling may prevent sufficient contact between the blood and additives, potentially contributing to incomplete inhibition of glycolysis. These two effects may explain why, in approximately 39% of the sample pairs, the glucose concentration in the NaF plasma samples was lower (mean difference = 5.1 ± 3.4 mg/dL, n = 10,554) than in the corresponding serum samples, which had already been centrifuged on time at the medical practice. On the other hand, if the serum was not centrifuged promptly, this may have led to a significant decrease in serum glucose concentration, resulting in lower serum than plasma glucose levels in approximately 61% of the paired samples (mean difference = 10.2 ± 7.9 mg/dL, n = 16,774). The difference exhibited an asymmetric distribution that varied with the mean glucose concentration. At glucose concentrations above 100 mg/dL, there was virtually no difference between the median serum and plasma glucose levels. In contrast, within the lower concentration range (below 100 mg/dL), a linear increase in the serum–plasma glucose difference was observed, with an increase of approximately 3.2 mg/dL for every 10 mg/dL decrease in mean glucose concentration. Bonetti et al. [24] reported a similar observation, finding that the difference between serum and plasma glucose increased by approximately 2.8 mg/dL for every 10 mg/dL decrease in the plasma glucose reference concentration after a four-hour incubation of the serum tube at room temperature. The reasons for the increasing difference between serum and plasma glucose with decreasing mean glucose concentrations below 100 mg/dL remain speculative. Since glycolysis in NaF tubes ceases a few hours after blood collection, the observed increase in the serum–plasma glucose difference can only be explained by an increased rate of glucose degradation in serum at lower basal glucose concentrations. This phenomenon cannot be readily explained by the enzyme kinetics underlying cellular glycolysis.

The key glycolytic enzymes hexokinase and phosphofructokinase-1 have very low Km values (Michaelis-Menten constants) and operate in the saturation range even at low glucose concentrations, meaning they metabolize glucose at a constant, maximal rate regardless of the basal glucose concentration [25]. Furthermore, glucose transporter type 1 (GLUT1), the transporter responsible for transmembrane glucose uptake into erythrocytes, operates at near-maximal capacity within the physiological range (80–100 mg/dL) and is driven exclusively by the glucose concentration gradient. Therefore, a reduction in extracellular glucose would be expected to decrease the absolute rate of glucose transport into erythrocytes [25]. A hypothetical explanation is that cellular stress responses triggered by glucose deprivation and oxidative stress may facilitate increased glucose consumption to support energy and redox homeostasis [25].

Our observation that the median difference between serum and plasma glucose was 0.5 to 3 mg/dL higher during the summer months (quarter 3) compared to the colder seasons (quarters 1, 2, and 4) can be attributed to the temperature dependence of the glycolysis rate, which increases at higher temperatures [4].

In summary, our comparative analysis of over 27,000 samples under routine conditions revealed only minimal differences between plasma and serum glucose concentrations above 100 mg/dL. As a result, at the diagnostic cutoff of 126 mg/dL, glucose measurement in plasma would identify only 0.3% more cases of diabetes mellitus than measurement in serum. This marginal benefit should be carefully weighed against the practical advantages of measuring glucose in serum in real-world outpatient settings. Serum is a universal sample matrix suitable for the analysis of numerous laboratory parameters [26], thereby avoiding the need for additional blood collection. A single blood draw is less stressful for patients and significantly reduces costs. Furthermore, it results in less plastic waste, contributing to environmental sustainability. Other studies have also shown that the use of serum for glucose measurement presents no disadvantages compared to NaF plasma, provided the serum is centrifuged and separated from blood cells in a timely manner after collection [8,21,27–29]. However, this requires that medical practices be equipped with centrifuges and that staff are properly trained by the laboratory in centrifugation procedures, as was the case in this study. If centrifugation is not feasible, the use of NaF plasma for glucose measurement is justified, with the caveat that glucose concentrations in NaF plasma also decrease by approximately 10 mg/dL within the first few hours after blood collection. Despite the good agreement observed between serum and NaF plasma glucose measurements at concentrations above 100 mg/dL, both specimen types must be regarded as suboptimal for the diagnosis of diabetes mellitus, as in vitro glycolysis is not sufficiently inhibited.

To achieve full reliability in glucose results by eliminating the preanalytical issue of glycolysis, a complete transition to citrated NaF/EDTA tubes would be necessary [20,22,24]. However, these tubes are not currently widely used in Germany, and a transition would be both costly and logistically challenging to implement in outpatient settings.

One of the main strengths of this study was the large dataset, comprising over 27,000 glucose measurements from concurrently collected serum and NaF plasma samples, all analyzed under routine laboratory conditions using the same measurement method over a three-year period. This enabled an assessment of the clinical relevance of the two sample types for glucose measurement and the diagnosis of diabetes mellitus in real-world outpatient settings.

The limitations of the study include the lack of information on pre-analytical variables such as the exact time of blood collection, the storage temperature of samples at the physician’s office prior to transport, the interval between blood collection and serum centrifugation, the time elapsed from collection to measurement, and the accuracy of mixing NaF samples after collection. Furthermore, no information was available regarding the indication for glucose measurement, patients’ clinical diagnoses, medication use, or other relevant clinical data. Consequently, it was not possible to evaluate potential correlations between glucose measurements and clinical diagnoses.

## Conclusions

This study, comprising more than 27,000 paired NaF plasma and serum samples collected over three years from outpatient practices, demonstrates that under real-world conditions, clinically relevant differences between serum and NaF plasma glucose concentrations were observed predominantly at glucose levels below 100 mg/dL. At concentrations above 100 mg/dL, the differences were minimal, such that at a diagnostic threshold of 126 mg/dL, only 0.3% more cases of diabetes mellitus would be identified based on NaF plasma compared with serum. Given the practical advantages of universally applicable serum tubes obtained from a single blood draw, this difference appears to be negligible for diabetes mellitus screening in routine outpatient settings. Serum may therefore be considered a pragmatic alternative to NaF plasma, provided that serum samples are centrifuged on-site in a timely manner using separator gel tubes.

Importantly, neither serum nor non-acidified NaF plasma provides complete and immediate inhibition of in vitro glycolysis. Accordingly, both specimen types must be regarded as suboptimal for the definitive biochemical diagnosis of diabetes mellitus when evaluated against current preanalytical standards. The present findings should therefore be interpreted as a comparison of two routinely used, but methodologically imperfect, sampling approaches under real-world outpatient conditions, rather than as an endorsement of their diagnostic adequacy.

Replacement of non-acidified NaF tubes with citrated NaF/EDTA tubes possibly alters these conclusions and would require re-evaluation.

## Supporting information

S1 Table Serum–plasma glucose differences by concentration quintiles and calendar quarters. (PDF)
