# Supplementary material for: Comparison of glucose concentrations in simultaneously collected plasma and serum samples from outpatients in a routine laboratory setting
Source: PLoS One. 2026 Mar 10;21(3):e0344562. doi: 10.1371/journal.pone.0344562 (PMC12974794; doi:10.1371/journal.pone.0344562)
Supplement: S1 Table — (PDF) [file pone.0344562.s001.pdf]

S1 Table. Serum–plasma glucose differences by concentration quintiles and calendar quarters. Shown are the medians and means of serum–plasma differences across quintiles of mean glucose concentration for the entire year (quarters 1–4) and for individual quarters. Glucose concentrations are expressed in mg/dL. **n**, number of observations; **CI**, confidence interval; **SD**, standard deviation.

Quintil		Quarter	n	Median	Median 95% CI	Mean	SD	Mean 95% CI
1	(41.3 - 77.8)	1-4	5,463	-11.05	-11.34 to -10.74	-11.40	10.10	-11.69 to -11.14
		1	1,633	-8.84	-9.44 to -8.34	-8.73	8.99	-9.17 to -8.29
		2	1,531	-11.50	-12.03 to -10.86	-12.02	10.63	-12.57 to -11.48
		3	1,380	-13.96	-14.66 to -13.30	-14.43	10.34	-14.98 to -13.87
		4	919	-9.89	-10.66 to -9.06	-10.63	9.47	-11.26 to -10.01
2	(77.8 - 84.5)	1-4	5,468	-5.20	-5.46 to -4.910	-5.61	8.80	-5.85 to -5.37
		1	1,704	-3.99	-4.54 to -3.59	-4.46	8.05	-4.85 to -4.07
		2	1,416	-4.46	-5.23 to -3.96	-5.23	9.09	-5.71 to -4.74
		3	1,266	-7.11	-7.72 to -6.30	-7.47	9.51	-8.00 to -6.93
		4	1,082	-5.66	-6.18 to -5.10	-5.76	8.32	-6.26 to -5.25
3	(84.5 - 91.5)	1-4	5,465	-1.69	-1.99 to -1.37	-2.80	8.55	-3.03 to -2.57
		1	1,654	-1.09	-1.55 to -0.44	-1.76	7.56	-2.13 to -1.38
		2	1,384	-0.74	-1.20 to -0.21	-2.41	8.92	-2.89 to -1.93
		3	1,220	-2.66	-3.39 to -1.93	-4.07	9.34	-4.61 to -3.54
		4	1,207	-2.74	-3.40 to -2.11	-3.39	8.38	-3.87 to -2.90
4	(91.5 - 106.2)	1-4	5,466	0.54	0.28 to 0.78	-1.09	8.49	-1.32 to -0.86
		1	1,699	1.41	0.93 to 1.76	-0.01	7.46	-0.38 to 0.35
		2	1,362	0.69	0.16 to 1.21	-1.07	8.95	-1.55 to -0.58
		3	1,165	-0.95	-1.69 to -0.41	-2.88	9.63	-3.44 to -2.31
		4	1,240	0.30	-0.33 to 0.89	-0.91	7.87	-1.36 to -0.47
5	(106.2 - 297.3)	1-4	5,466	1.13	0.93 to 1.41	-0.64	9.31	-0.89 to -0.39
		1	1,870	2.00	1.55 to 2.35	0.41	8.21	0.03 to 0.79
		2	1,448	1.09	0.62 to 1.64	-0.71	9.61	-1.22 to -0.21
		3	1,055	-0.79	-1.45 to -0.07	-2.98	10.87	-3.65 to -2.31
		4	1,093	1.31	0.61 to 1.91	-0.06	8.65	-0.58 to 0.47
